# Intestinal Immunomodulation and Shifts on the Gut Microbiota of BALB/c Mice Promoted by Two* Bifidobacterium* and* Lactobacillus* Strains Isolated from Human Samples

**DOI:** 10.1155/2019/2323540

**Published:** 2019-04-18

**Authors:** Alicja M. Nogacka, Sofia Oddi, Nuria Salazar, Jorge A. Reinheimer, Miguel Gueimonde, Gabriel Vinderola, Clara G. de los Reyes-Gavilán

**Affiliations:** ^1^Department of Microbiology and Biochemistry of Dairy Products, Instituto de Productos Lácteos de Asturias (IPLA-CSIC), 33300 Villaviciosa, Asturias, Spain; ^2^Instituto de Lactología Industrial (INLAIN, UNL-CONICET), Facultad de Ingeniería Química, Universidad Nacional del Litoral, Santiago del Estero 2829, Santa Fe 3000, Argentina; ^3^Diet, Microbiota and Health Group, Instituto de Investigación Sanitaria del Principado de Asturias (ISPA), Spain

## Abstract

*Bifidobacterium animalis* subsp.* lactis *IPLA 20020 and* Lactobacillus gasseri* IPLA 20212, two strains isolated from human samples, were evaluated for safety and influence over the intestinal microbiota and cytokine production by the intestinal tissue of adult BALB/c mice. Mice were divided into four groups receiving during 8 days PBS or a suspension of each strain, prepared fresh or lyophilized (bifidobacteria), at an amount of 4x10^8^ viable cells/day. This dose could be comparable to the probiotic intake of a human adult who consumed about 100-200 mL of functional fermented milk per day, considering the usual level of probiotics in commercial products. No microbial translocation to liver or alterations in food intake, weight, and behavior were observed in treated mice. Intestinal content of secretory immunoglobulin A (s-IgA) was not affected, discarding any adverse effect on the mucosa-associated immunity. The profile of intestinal proinflammatory/regulatory cytokines after intervention evidenced that the microbial strain administered and its cellular state (fresh or lyophilized) as well as the host tissue analyzed (small or large intestine) influenced the immune response and suggests a moderate shift towards a T helper 1 profile (Th1) in the large intestine after the administration of both strains. Changes on relative levels of some intestinal microbial groups were evidenced after intervention. It is noteworthy that butyrate was positively associated with a balanced pro-Th1 immune response. Therefore,* B. animalis* subsp*. lactis *IPLA20020 and* L. gasseri* IPLA 20212 could be considered potential probiotic candidates to be included in functional foods for balancing the intestinal immune response.

## 1. Introduction

Probiotics are defined as live microorganisms that when administered in adequate amounts confer a health benefit to the host [1]. Although they are usually administered included in food, some lyophilized preparations are also available in the market as supplements [2]. The beneficial effects attributed to probiotics are strain specific and require demonstration in expensive and time-consuming human intervention studies, which frequently fail to confirm previous* in vitro* observations [3]. The use of animal models is an affordable intermediate step, recommended for the selection of suitable probiotics [1].

Microorganisms from* Lactobacillus* and* Bifidobacterium* genera constitute the most frequently used human probiotics, being relatively easy to produce them in large scale as frozen or freeze-dried cultures [4]. Due to their long history of safe use, some of their species have the “Qualified Presumption of Safety” (QPS) status recognized by the European Food Safety Authority (EFSA) [5]. Breast milk and fecal samples of newborns represent a good source of potential probiotics from the genera* Bifidobacterium* and* Lactobacillus* [6, 7]. The gastrointestinal tract (GIT) is the first line of action of probiotics, where they can interact directly with cells from GIT epithelium and cells from the immune system and they can modulate the intestinal microbiota composition and their subsequent interaction with the host [8]. Therefore, the knowledge of the immune modulation profile of potential probiotics at the intestinal level could facilitate the selection of strains tailored for their specific immune properties and safety for human consumption in foods [9].

FAO/WHO [10] recommends intervention studies in animals, mainly rodents, as a previous step to the human intervention studies definitely proving functionality of probiotics. BALB/c is the most frequently used mice strain to study functionality and mechanisms of action of probiotics and has been recommended for the assessment of the immunomodulatory capacity of lactic acid bacteria [11].* Bifidobacterium animalis* subsp.* lactis* IPLA 20020 and* Lactobacillus gasseri* IPLA 20212 are two strains previously isolated by us from infant feces and breast milk, respectively [7]. The aim of the present study was to perform a preliminary evaluation of these strains as potential probiotics by determining their safety and influence on the pattern of gut cytokines and on the intestinal microbiota profile of adult BALB/c mice.

## 2. Materials and Methods

### 2.1. Microorganisms and Growth Conditions

Microbial strains were reactivated from frozen stocks by overnight incubation at 37°C in MRS broth (Biokar Diagnostics, Beauvais, France), being added with 0.1% (v/v) L-cysteine (Sigma Chemical Co., St Louis, MO, USA) for the cultivation of* Bifidobacterium*. In the case of the bifidobacteria, incubation was carried out in anaerobic conditions, as previously specified [7]. For the preparation of strains to be administered to experimental animals, MRS broth was inoculated (1% w/v) with the corresponding overnight microbial cultures and then pellets were washed with sterile PBS and resuspended in the same buffer. In order to determine whether the format of administration of strains could influence the results obtained, part of the* B. animalis* subsp.* lactis* suspension was also freeze-dried; with this aim, pelleted overnight cultures were resuspended in 10% (w/v) sucrose, kept at -80°C in sterilized glass tubes for 24 h and then lyophilized in a Freezemobile 12EL equipment (VirTis, Gardiner NY, USA) for 24 h. Fresh inocula of* Bifidobacterium *and* Lactobacillus* strains as well as the lyophilized bifidobacteria were prepared at a concentration of 2x10^9^ cfu/mL in PBS and were administered to mice.

### 2.2. Animals and Experimental Design

The experiments with animals were approved by the Ethical Committee for Animal Experimentation of the Facultad de Ciencias Veterinarias, Universidad Nacional del Litoral (Esperanza, Santa Fe, Argentina). Subsequent handling strictly followed the European Communities Council Directive of November 24, 1986 (86/609/EEC). Twenty-one six-week-old male BALB/c mice (22 ± 1 g body weight) were obtained from the Random Bred Colony at the Centro de Medicina Comparada, Instituto de Ciencias Veterinarias del Litoral (CMC-ICiVet-Litoral, CONICET–UNL), Facultad de Ciencias Veterinarias, Universidad Nacional del Litoral (Esperanza, Santa Fe, Argentina) and were maintained at the INLAIN animal facility for 7 days before the trial began. Animals were kept in a controlled environment at a temperature of 21 ± 2°C with humidity at 55 ± 2% and 12 h light-dark cycle during all the intervention. All animals received* ad libitum* sterile tap water and a sterile high-protein and normal carbohydrate balanced diet (Cooperación, Buenos Aires, Argentina) containing per kg 320 g carbohydrates, 230 g proteins, 75 g total fat, 60 g raw fiber, 100 g total minerals, 13 g Ca, 8 g P, 120 g water, and vitamins. This dietary profile is routinely used in our laboratory for optimal metabolic performance of mice, following the US Department of Agriculture (USDA) recommendations [12].

Mice were simultaneously divided into four experimental groups of 5-6 animals each and housed in plastic cages. Animals received daily by oral gavage during 8 days 0.2 mL of PBS solution with different bacterial preparations, as follows: group (1) fresh* B. animalis* subsp.* lactis* IPLA 20020, group (2) lyophilized* B. animalis* subsp.* lactis,* group (3) fresh* L. gasseri* IPLA 20212, and group (4) control: no microorganism administered. Feces from mice were collected from cages at day 0 and day 8 of intervention, immediately frozen at -80°C, and freeze-dried. Weight of animals, food consumption, and mortality were monitored daily; animals were also observed daily for possible abnormal behavior. At the end of the intervention period, animals were anesthetized individually by an intraperitoneal injection of a ketamine-xylazine-acepromazine cocktail. Animals were sacrificed by cervical dislocation. The liver and small and large intestines were aseptically excised from dead animals. Intestinal fluid was obtained by flushing the small intestine with 5 mL of PBS [13] added with a protease inhibitor cocktail (Sigma P8340) (1% v/v). Intestinal fluid and small and large intestine tissue samples were kept at -70°C until processing whereas liver samples were processed immediately.

### 2.3. Liver Cultivation

Liver samples were suspended immediately after collection in 1/10 sterile PBS solution and homogenized (Ultra Turrax T8, Ika Labortechnik, Staufen, Germany) for 30 seconds; serial dilutions in PBS buffer were deep-plated into VRBL agar (Biokar, Beauvais, France) and incubated 24 h at 37°C for enterobacteria counting to assess intestinal bacterial translocation.

### 2.4. Determination of Intestinal Immune Parameters

Intestinal fluid and small and large intestine tissue samples were kept at -70°C until processing for secretory IgA (s-IgA) and cytokine determination. Cytokines (TNF*α*, IFN*γ*, IL-10, IL-6, IL-12, and IL-2) were determined by ELISA in intestinal tissue homogenates, using commercial kits (BD Biosciences Pharmingen, San Diego, CA, USA). For preparing homogenates, intestinal tissue samples were suspended in PBS containing 1% (v/v) antiprotease cocktail (Sigma), 10 mM EDTA (Sigma), and 0.05% (v/v) Tween 20 (Sigma) in a proportion of 1 mL PBS per 100 mg tissue and were homogenized for 30 seconds (Ultra Turrax T8) and centrifuged (9500x*g*, 10 min, 4°C) and the supernatants were collected and kept frozen until use. S-IgA was quantified by ELISA in intestinal fluids collected as indicated before [14].

### 2.5. Analysis of Microbial Communities in Fecal Samples

Assessment of fecal microbial groups was made by qPCR. The DNA was obtained from lyophilized fecal samples diluted 1/10 in PBS by using the QIAamp DNA stool mini kit (Qiagen, Hilden, Germany). PCR amplification of the 16S rRNA gene for the quantification of different bacterial groups (*Bacteroides–Prevotella–Porphyromonas *group*, Bifidobacterium, Akkermansia, Clostridium *cluster XVIa*, Lactobacillus *group, and* Faecalibacterium*) and levels of total intestinal bacteria was performed in a 7500 Fast Real-Time PCR System (Applied Biosystems, Foster City, CA, USA) using the SYBR Green PCR Master Mix (Applied Biosystems) as described previously [15]. Samples were analyzed in duplicate in two independent PCR runs. Data of lyophilized fecal samples were normalized for comparison. In this way, the relative abundances of the different microbial groups were calculated as a percentage of the total microbial levels in each sample; percentage of variations of the relative abundance for each microbial group after 8 days of intervention were then referred to as the relative abundance of this group at time 0 considering this as 100%.

Analysis of the major short chain fatty acids (SCFA: acetic, propionic, and butyric acids) was performed in a 1/10 PBS dilution of the lyophilized fecal sample. A gas chromatograph 6890N (Agilent Technologies Inc, Palo Alto, CA, USA) connected to a mass spectrometry (MS) 5973N detector (Agilent Technologies) and to a flame ionization detector (FID) was used as described previously [16]. Samples were analyzed in triplicate and the molar proportion of each SCFA was calculated as the concentration (mM) percentage of such compound referred to as the total SCFA (sum of concentrations of the three major SCFA: acetic, propionic, and butyric acids). Increments in the molar proportion of each of the major SCFA at the end of the intervention (day 8) were calculated with respect to the initial molar proportion for each compound at time 0.

### 2.6. Statistical Analyses

Statistical analysis of results was performed using the software SPSS v.24 (SPSS Inc., Chicago, USA). Data were compared among the different groups of mice administered probiotics and the control group at the end of the intervention. For variables showing a normal distribution (Shapiro-Wilk and Levene tests), one-way ANOVA followed by post hoc DMS comparison was conducted (increments of acetic, propionic, and butyric acids, acetic to propionic and acetic to butyric acids ratios in feces; s-IgA in intestinal fluid; IL-10, Il-6, and IFN*γ* in the small intestine tissue; TNF*α*, and IL-6 in the large intestine tissue). In the remaining cases (variables showing nonnormal distribution) a Kruskal-Wallis test and a Dunn's test of pairwise comparisons were applied. To get deep into the associations between the microbiota and the cytokines in the large intestine tissue, a Spearman correlation analysis was conducted considering together all the animals of our study. A heatmap was generated under R Studio version 1.1.456 package heatmaply. A significant* p* value of 0.05 was used for the interpretation of results.

## 3. Results and Discussion

The amount of bacteria administered to mice in our study is comparable to the dose of probiotic received per unit of body weight by a human adult consuming 100-200 mL of fermented milks containing about 10^9^ viable probiotics per mL. Safety is one of the most important criteria for the selection of probiotics. No death or unexpected changes in food intake or behavior were observed for any of the animal groups receiving* Bifidobacterium* and* Lactobacillus *strains and no statistically significant differences in the animal weight were noticed among the four groups of mice during the treatment (data not shown). This indicates that general parameters of well-being were not affected by the administration of* L. gasseri* IPLA 20212 and* B. animalis* subsp.* lactis* IPLA 20020 strains to BALB/c mice. In addition, no microbial counts were obtained after the intervention from the cultivation of liver tissue, one of the first reliable signals of microbial translocation, evidencing that the oral administration of the microorganisms did not promote bacterial translocation. Therefore, no adverse systemic effects were noticed by the administration of the two microbial strains to mice at levels comparable with those of human consumers of fermented milks containing probiotics.

S-IgA is the most abundant antibody in the mucosal secretions and plays a key role in the defense against pathogens. S-IgA levels determined in the intestinal fluid did not show any significant variation between the groups of mice after the period of administration of the* Bifidobacterium* and* Lactobacillus* strains (mean ± standard deviation: 11.23 ± 2.39 *μ*g/mL in the control group, 8.96± 1.53 *μ*g/mL in mice fed fresh* Bifidobacterium*, 11.57 ± 2.85 *μ*g/mL in mice fed lyophilized* Bifidobacterium*, and 10.57 ± 4.40 in mice fed* Lactobacillus*). These results indicate that s-IgA is being produced similarly by all groups of animals, allowing us to rule out possible adverse effects on the mucosa-associated immunity by the microorganisms administered.

**Figure 1:**
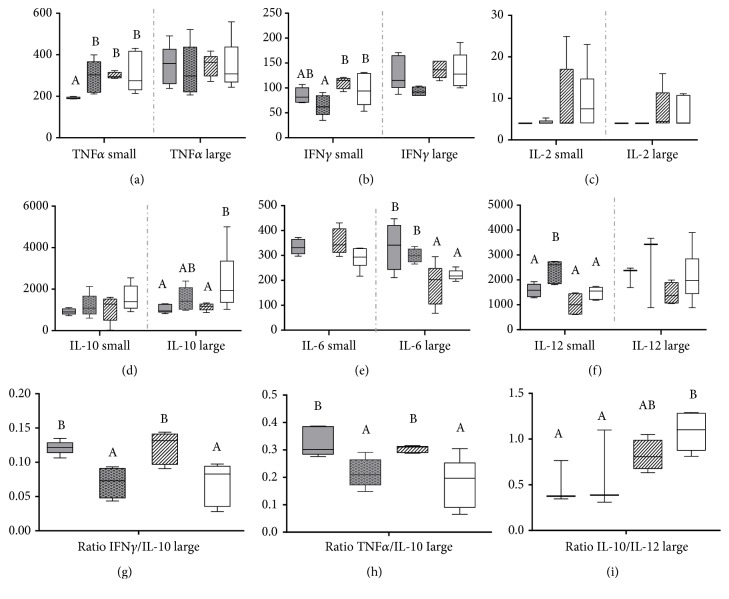
Cytokines ((a) to (f)) (pg/mL) in tissue of small intestine (left part of graphs) and large intestine (right part of graphs) and cytokine ratios in the large intestine ((g) to (i)) of BALB/c mice after a daily administration during 8 days of the vehicle (PBS) (white boxes), fresh* B. animalis* subsp.* lactis* IPLA 20020 (grey boxes), lyophilized* B. animalis* subsp.* lactis* IPLA 20020 (dotted grey boxes), and fresh* L. gasseri* IPLA 20212 (striped white boxes). For each cytokine and cytokines ratio, the box and whiskers plot represents median, interquartile range, and minimum and maximum values obtained from each group of animals. A one-way ANOVA test and a DMS post hoc test were used to compare IL-10, IL-6, and IFN*γ* in the small intestine and TNF*α* and IL-6 in the large intestine tissues among the four groups of mice whereas the nonparametric Kruskal-Wallis and pairwise comparison with Dunn's tests was applied to the comparison of TNF*α* in the small intestine tissue, INF*ϒ* and IL-10 in the large intestine tissue, and IL-2, IL-12 and TNF*α*/IL-10, IFN*γ*/IL-10, and IL10/IL-12 ratios in the small and large intestine tissues among the four groups of animals. Different letters above the boxes indicate significant differences in small intestine, large intestine, and cytokine ratios in the large intestine, among the four groups of animals receiving different probiotics or vehicle (*p* < 0.05).

We determined the levels of different cytokines in tissue homogenates of small and large intestine from the four groups of mice after the treatments (Figures 1(a)–1(f)). The effects promoted by the administration of* B. animalis* subsp.* lactis* IPLA 20020 or* L. gasseri* IPLA 20212 were moderate, differed between the small and large intestine tissues, and varied depending on the microbial strain administered and its cellular state. In this respect, it has been previously reported that different strains may promote different responses, inducing even opposite immune effects [17, 18]. Specifically, in the present work we found differences among mice groups for TNF*α*, IFN*γ*, and IL-12 in the small intestine tissue whereas in the large intestine differences for IL-10 and IL-6 were found. Regarding the small intestine, the administration of a fresh culture of* B. animalis* subsp.* lactis* IPLA 20020 promoted lower levels of TNF*α* whereas the lyophilized preparation of the same microorganism increased IL-12 with respect to the control group; regarding the IFN-*γ*, only the lyophilized preparation of the bifidobacteria was able to promote a decrease in the levels of this cytokine. In the large intestine tissue of mice fed either* B. animalis* subsp.* lactis* IPLA 20020 or* L. gasseri *IPLA 20212, the levels of IL-10 decreased significantly regarding the control group, which was accompanied by a concomitant increase of IL-6 in animals receiving the* Bifidobacterium* strain (either administered fresh or lyophilized).

Since cytokines do not act in an isolated way, the predominant type of immune response depends on the balance between different cytokines. High levels of IL-10 with respect to IL-12 may redirect the immune response towards a type Th2 or a T regulatory response. Moreover, IL-10 and TNF*α* are cytokines mutually regulated and with opposite roles in inflammation, so that their relative balance is important for controlling deviations on the immune responses. Therefore, several ratios between cytokines that are relevant for dendritic cell induced T lymphocyte differentiation responses were calculated to predict the Th-cell type of predominant response induced in the intestinal tissue of mice by the administration of our microbial strains (IL-10/IL-12, TNF*α*/IL-10, and IFN*γ*/IL-10) (Figures 1(g)–1(i)). In this respect, it is also interesting to consider that BALB/c mice naturally display a Th1/Th2 balance towards Th2 response [19], which makes these animals useful for assessing shifts towards Th1 responses promoted by different agents. Regarding our results, in the large intestine, a moderate but statistically significant increase of the TNF*α*/IL-10 and IFN*γ*/IL-10 ratios occurred following the administration of fresh cultures of either the two strains whereas the bifidobacteria (either fresh or lyophilized) promoted a decrease of the IL-10/IL-12 ratio. Therefore it is reasonable to expect that both strains promote a moderate shift towards a Th1 response, which in the case of the* Bifidobacterium *strain could be regulated by the concomitant increase in IL-6 levels. The effect promoted in the small intestine by the administration of* B. animalis* subsp.* lactis* IPLA 20020 and* L. gasseri* IPLA 20212 is less conclusive, as the variations on cytokines did not result in any significant change in cytokine ratios that could suggest a modulation of the immune response (data not shown). A similar pattern of immune modulatory profile, with a moderate enhancement of the production of proinflammatory cytokines and a cytokine balance favoring Th1 cellular immunity, has been previously reported for some* Lactobacillus* and* Bifidobacterium* strains in rodent models [20–22]. Early infancy is characterized by an immature and Th2-biased immune system; the postnatal T helper cell maturation and functionality are mainly driven by the interaction between the gut associated lymphoid tissue and microbiota, tending to favor a Th1 polarization and the subsequent Th1-Th2 homeostasis [23, 24]. Considering that the two strains tested in our study were isolated in the neonatal period (from feces of a 2-day-old full-term vaginally delivered breast-fed infant and from breast milk, for* B. animalis* subsp.* lactis* and* L. gasseri *strains, respectively), this could be related with the Th1-biased immunity promoted by these microorganisms on the intestinal tissue of mice [23].

Interestingly, our results indicate that the form of administration of microorganisms may influence the final results obtained. In this regard, some authors have previously indicated that technological processes and cellular state can modify the immunomodulating capability of probiotic bacteria [25–27].

In a next step we wanted to know whether the administration of the strains affected the intestinal microbiota. Thus, the relative levels with respect to the total microbial counts of some relevant intestinal microbial groups were determined before and after the administration of* B. animalis *subsp.* lactis* IPLA 20020 or* L. gasseri* IPLA 20212 (Table S1). Counts of* Akkermansia*, below the detection limit of the technique (< 3 log n° cells/gram), and the low levels obtained for* Faecalibacterium* as compared to total bacteria prevented extracting any conclusion for these two microbial groups (data not shown). We further determined the percentage of variation within each group of mice of the intestinal microbial groups at the end of treatment (with respect to time 0) and then compared these values among mice receiving different treatments (Table 1). The fecal levels of* Lactobacillus*-*Weissella *decreased in control mice along the intervention but increased in animals treated with* B. animalis* subsp.* lactis* (administered either fresh or lyophilized) and* L. gasseri*; this resulted in significantly higher levels of* Lactobacillus*-*Weissella *at the end of the intervention for those mice receiving* B. animalis* or* L. gasseri*. On the other hand,* Enterobacteriaceae* increased along intervention both in control mice and in those administered* Bifidobacterium* whereas animals receiving* L. gasseri* experienced a decrease of this intestinal microbial group, which gave rise to significantly lower levels of enterobacteria at the end of the intervention in mice receiving* L. gasseri* with respect to the other groups of mice.* Lactobacillus* is among the most abundant intestinal microorganisms in mice [28] and its increase together with decreases in* Enterobacteriaceae* has been related with beneficial health parameters in murine models displaying dysbiosis associated to diseases [29–31]. No differences or clear trends were observed for the other fecal microbial groups analyzed. Moreover, molar proportions of fecal SCFA were calculated at time 0 and after 8 days of treatment (Table S2). No significant differences in the variations of molar proportions of acetic, propionic, and butyric acids as well as in the acetic to propionic and acetic to butyric acid ratios were found at the end of the treatment with respect to time 0, when comparing the different groups of mice (data not shown).

**Figure 2:**
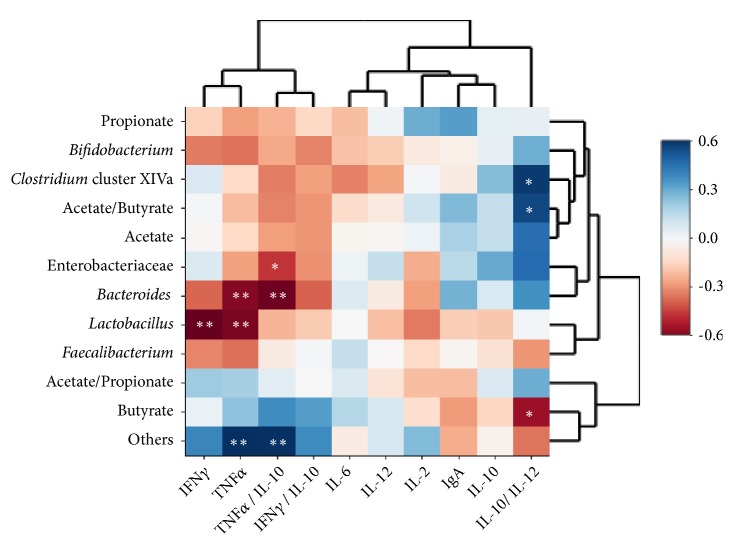
Spearman correlation between cytokines in the large intestine tissue and fecal microbiota of the whole mice population used in this work after 8 days of administration of vehicle (PBS),* B. animalis* subsp.* lactis* IPLA 20020 (fresh or lyophilized) and fresh* L. gasseri* IPLA 20212. Columns correspond to fecal SCFA (molar proportions), fecal SCFA ratios, and relative abundance of intestinal microbial groups (Table S1); rows correspond to cytokines and some relevant ratios among them. Blue and red colors denote negative and positive associations, respectively. The intensity of the colors represents the degree of association between cytokines determined in the large intestine and the intestinal microbiota and SCFA. Asterisks indicate significant associations: *∗p* < 0.05; *∗∗ p* < 0.01.

We further looked for possible associations between immune parameters and the microbiota at the end of the intervention period in the whole set of animals used in this work. The IL-10/IL-12 ratio associated inversely with the intestinal molar proportions of butyric acid and directly with the acetic to butyric acids ratio (Figure 2). Butyric acid is an essential metabolite in the colon, being the main energy source for colonocytes, contributing to the maintenance of the gut barrier and acting, as well as other SCFA, on the innate and adaptive immune responses [32]. A moderate increase of butyric acid concentrations and a decrease of the acetic to butyric acids ratio have been generally considered beneficial for adult's health [33] whereas in infants a delayed shift in the production of butyric acid could reflect an altered intestinal colonization [6]. We also found an inverse association between the proinflammatory cytokines TNF*α* and IFN*γ* with some of the fecal microbial groups (mainly* Lactobacillus* and* Bacteroides-Prevotella-Porphyromonas*); interestingly, the IL-10/IL-12 ratio appears to be associated with* Clostridium* cluster XIVa, a group known as butyric acid producer, whereas the ratio TNF*α*/IL-10 directly correlated with other unknown members of the intestinal microbiota. These associations suggest the involvement of other intestinal microorganisms not analyzed in the present work, directly or through interactions with other members of the intestinal microbiota, in the production of butyric acid.

The potential probiotic strains used in the present study could be applied in infants presenting a delayed Th1 polarization of the immune response or in adults having weakened Th1 response. Yet, both species studied are considered to possess core benefits, according to the approach presented by Hill et al. [1]. Health Canada, for instance, incorporated this approach to their legal frame and acknowledges probiotic properties to any strain belonging to the species of microorganisms used in this study when delivered in food at a level of 1x10^9^ cfu per serving. Acceptable claims on Canadian foods for these probiotics are based on their contribution to a healthy gut microbiota. Further studies aimed at analyzing more in depth the effects of* B. animalis* subsp.* lactis* IPLA 20020 and* L. gasseri* IPLA 20212 on the immune system and on the intestinal microbiota need to be addressed in order to know the potential applicability of these strains to be included in fermented milks or other functional food products as well as the human groups to which they could be applied.

## 4. Conclusion

*Bifidobacterium animalis* subsp.* lactis *IPLA 20020 and* Lactobacillus gasseri* IPLA 20212, when administered to adult male BALB/c mice, promoted a moderate shift towards a Th1 immune response profile in the large intestine tissue, which positively associated with fecal levels of butyrate and with changes in the relative levels of some relevant fecal microbial groups. These effects were dependent on the strain and, in the case of the bifidobacteria, on the format of administration of the microorganism to animals (solutions prepared from fresh or lyophilized cultures). Our results indicate the potentiality as probiotics of the two strains tested and highlight the impact of the format of probiotic administration on the* in vivo* functionality.

## Figures and Tables

**Table 1:** Percentage of variation (Δ) with respect to time 0 (considered as 100%) of the relative abundance of different intestinal microbial groups (Table S1) after eight days of daily administration to adult BALB/c mice of vehicle (PBS), *B. animalis* subsp. *lactis* IPLA 20020 (fresh or lyophilized), and fresh *L. gasseri* IPLA 20212. “Others” refers to the rest of microbial groups not determined in the present work. The nonparametric Kruskal-Wallis and pairwise comparison with Dunn's test were used to compare the four groups of mice. Different letters in superscript indicate significant differences among groups of animals receiving different probiotics or placebo (*p* < 0.05).

Microbial groups	Control	*B. animalis* subsp. *lactis *IPLA 20020	*B. animalis* subsp. *lactis *IPLA 20020 lyophilized	*L. gasseri* IPLA 20212
Δ *Bacteroides-Prevotella-Porphyromonas *	-0.59 ± 18.00	-13.51 ± 17.19	-14.47 ± 25.41	-13.20 ± 12.80
Δ *Bifidobacterium*	33.84 ± 58.36	11.16 ± 38.27	49.26 ± 96.98	39.11 ± 63.89
Δ *Lactobacillus-Weissella *	-57.31 ± 32.60^a^	183.35 ± 355.62^ab^	271.12 ± 309.88^b^	353.10 ± 483.71^b^
Δ Enterobacteriaceae	262.40 ± 232.18^b^	169.54 ± 262.98^b^	63.63 ± 101.26^ab^	-40.26 ± 31.67^a^
Δ *Clostridium* cluster XIVa	219.30 ± 320.62	-18.02 ± 72.05	33.07 ± 172.02	-18.11 ± 23.91
Δ Others	-10.54 ± 144.45	24.83 ± 29.66	171.26 ± 341.33	30.65 ± 34.65

## Data Availability

Data used to support the findings of this study are included within the article and supplementary material.

## Supplementary Materials

Supplementary Materials Table S1: relative abundance of different intestinal microbial groups after the daily administration of* Lactobacillus* and* Bifidobacterium* strains to adult BALB/c mice. Table S2: molar proportions of the major short chain fatty acids after the daily administration of* Lactobacillus* and* Bifidobacterium* strains to adult BALB/c mice.
