# Supplementary material for: Intestinal Immunomodulation and Shifts on the Gut Microbiota of BALB/c Mice Promoted by Two Bifidobacterium and Lactobacillus Strains Isolated from Human Samples
Source: Biomed Res Int. 2019 Apr 18;2019:2323540. doi: 10.1155/2019/2323540 (PMC6500685; doi:10.1155/2019/2323540)
Supplement: Supplementary Materials — Table S1: relative abundance of different intestinal microbial groups after the daily administration of Lactobacillus and Bifidobacterium strains to adult BALB/c mice. Table S2: molar proportions of the major short chain fatty acids after the daily administration of Lactobacillus and Bifidobacterium strains to adult BALB/c mice. [file 2323540.f1.pdf]

Supplementary Material

Table S1. Relative abundance of different intestinal microbial groups, calculated as a percentage (mean ±standard deviation) of the total microbial counts, at time 0 and after eight days of daily administration of *B. animalis* subsp. *lactis* IPLA 20020 (fresh culture or lyophilized) or *L. gasseri* IPLA 20212 (fresh culture), and a placebo (control) to adult BALB/c mice. Total counts at each sampling time are indicated as log nº cells/g

Intestinal microbial groups	Control		*B. animalis* subsp. *lactis* fresh		*B. animalis* subsp. *lactis* lyophilized		*L. gasseri* fresh	
	0 days	8 days	0 days	8 days	0 days	8 days	0 days	8 days
Bacteroides-Prevotella-Phorphyromonas group	77.620 ± 11.895	77.709 ± 19.905	60.747 ± 4.680	52.218 ± 8.685	78.480 ± 9.499	65.675 ± 15.705	62.230 ± 9.409	53.181 ± 4.215
Bifidobacterium	0.634 ± 0.105	0.875 ± 0.468	0.325 ± 0.044	0.355 ± 0.108	0.651 ± 0.202	0.947 ± 0.679	0.455 ± 0.107	0.598 ± 0.166
Lactobacillus-Weisella group	0.899 ± 0.666	0.527 ± 0.681	0.286 ± 0.286	0.436 ± 0.518	0.478 ± 0.278	1.123 ± 0.505	0.139 ± 0.074	0.414 ± 0.305
Enterobacteriaceae	0.007 ± 0.003	0.026 ± 0.018	0.003 ± 0.001	0.007 ± 0.005	0.003 ± 0.001	0.005 ± 0.003	0.005 ± 0.003	0.003 ± 0.002
Clostridium cluster XIVa	1.145 ± 0.633	2.404 ± 1.106	0.702 ± 0.484	0.382 ± 0.331	1.053 ± 0.357	1.182 ± 1.534	0.502 ± 0.188	0.414 ± 0.073
Others	19.694 ± 12.904	18.459 ± 21.658	37.937 ± 4.699	46.602 ± 9.022	19.335 ± 9.966	31.070 ± 18.237	36.669 ± 9.641	45.473 ± 4.354
Total counts	11.216 ± 0.244	10.975 ± 0.381	11.463 ± 0.156	11.534 ± 0.094	11.438 ± 0.121	11.250 ± 0.238	11.505 ± 0.069	11.433 ± 0.087

Table S2. Molar proportions of the major SCFA (acetate, propionate, and butyrate), calculated as a percentage of the total SCFA (sum of concentrations of the three major SCFA expressed in mM) at time 0 and after eight days of daily administration of *B. animalis* subsp. *lactis* IPLA 20020 (fresh culture or lyophilized) or *L. gasseri* IPLA 20212 (fresh culture), and a placebo (control) to adult BALB/c mice. Ratios between some SCFA are also indicated.

Intestinal SCFA	Control		*B. animalis* subsp. *lactis* fresh		*B. animalis* subsp. *lactis* lyophilized		*L. gasseri* fresh	
	0 days	8 days	0 days	8 days	0 days	8 days	0 days	8 days
Acetic acid	76.35 ± 2.09	78.08 ± 2.48	75.37 ± 3.79	70.47 ± 3.83	75.55 ± 1.09	72.96 ± 3.43	73.15 ± 1.99	72.12 ± 3.31
Propionic acid	15.15 ± 2.01	14.22 ± 1.16	12.58 ± 1.75	12.84 ± 1.1	14.08 ± 0.76	13.59 ± 1.46	13.3 ± 2.51	14.81 ± 1.32
Butyric acid	8.5 ± 1.49	7.7 ± 1.63	12.05 ± 2.46	16.68 ± 4.27	10.38 ± 1.18	13.45 ± 3.77	13.56 ± 2.16	13.07 ± 2.44
Acetic/Propionic	5.11 ± 0.69	5.53 ± 0.63	5.69 ± 0.71	5.52 ± 0.49	5.38 ± 0.31	5.41 ± 0.6	5.68 ± 1.22	4.91 ± 0.63
Acetic/Butyric	9.27 ± 2.06	10.53 ± 2.23	6.52 ± 1.61	4.58 ± 1.77	7.36 ± 0.86	5.81 ± 1.77	5.54 ± 1.12	5.72 ± 1.38
Total SCFA (mM)	95.56 ± 23.53	66.99 ± 23.07	131.27 ± 36.30	109.54 ± 26.68	128.13 ± 23.53	112.40 ± 30.74	156.04± 26.72	137.72 ± 32.20
